# Shenfu Decoction Extends Survival Time of Seawater-Induced Hypothermia in Rats: The Role of Metabolomics and Gut Microbiota

**DOI:** 10.2174/0113892002403722251122095723

**Published:** 2026-01-20

**Authors:** Ya-jing Wang, Hong-zhi Chen, Zhi-bo Wang, Chao-yue Sun, Chen-yang Guo, Yi Ruan, Chuan-tao Li, Bin Zou, Zi-fei Yin, Wei Gu

**Affiliations:** 1 School of Traditional Chinese Medicine, Naval Medical University, Shanghai 200433, China;; 2 Yueyang Hospital of Integrated Traditional Chinese and Western Medicine, Shanghai University of Traditional Chinese Medicine, Shanghai, 200433, China;; 3 Basic Medicine College, Naval Medical University, Shanghai, 200433, China;; 4 Department of Traditional Chinese Medicine, PLA Naval Medical Center, Shanghai, 200052, China;; 5 Naval Medical Center of Naval Medical University Aviation Physiological and Psychological Training Forces, Shanghai, 200433, China;; 6 Dujiangyan Air Force Special Service Sanatorium, Chengdu, 611838, China;; 7 Department of Biochemistry and Molecular Biology, College of Basic Medical, Naval Medical University, Shanghai, 200433, China;; 8 Department of Rehabilitation, Changhai Hospital, Naval Medical University, Shanghai, 200433, China

**Keywords:** Shenfu decoction, survival time, seawater immersion hypothermia, gastrointestinal microbiome, metabolomics, thermogenesis

## Abstract

**Introduction:**

Shenfu decoction (SFD), a Traditional Chinese Medicine formula, is used in clinical emergencies. Its effects on seawater-induced hypothermia remain unclear. This study investigates the therapeutic mechanisms of SFD in improving the survival of hypothermic rats through metabolomics and gut microbiota analysis.

**Methods:**

Hypothermia was induced in rats *via* seawater immersion. The chemical constituents of SFD were analyzed using ultra-performance liquid chromatography quadrupole time-of-flight mass spectrometry (UPLC-Q-TOF-MS). Survival time and rates of low-temperature water-immersed rats were assessed. Rat blood samples were obtained for analysis of hematologic parameters, electrolytes, hepatic and renal function, cardiac injury, and inflammatory cytokines. To investigate the potential mechanism underlying the survival-prolonging effect of SFD on seawater-immersed hypothermic rats, untargeted blood metabolomics and gut microbiota profiling were employed for preliminary screening.

**Results:**

UPLC-Q-TOF-MS identified almost 50 compounds in SFD, and 1.35 g/kg SFD significantly extended the survival time of seawater-induced hypothermia rats by 6 hours. After hypothermic seawater immersion, the levels of red blood cells, hemoglobin, hematocrit, as well as serum calcium, phosphorus, blood urea nitrogen, alkaline phosphatase, total protein, cardiac troponin T, and interleukin-6 were significantly increased. However, pretreatment with 1.35 g/kg SFD in rats markedly decreased these parameters. The induction of hypothermic seawater immersion elevated blood glucose, and the administration of SFD exacerbated this increase in rats. Metabolomic analysis revealed elevated levels of valerenic acid and benzoylmesaconine in the SFD group, suggesting the restoration of metabolic homeostasis. This recovery was associated with modulation of the gut microbiota, notably an enhancement of beneficial genera, such as Enterococcus.

**Discussion:**

The findings demonstrated that SFD significantly prolonged survival in a rat model of seawater-immersion hypothermia. The protective mechanism involved a dual action: mitigating hypothermia-induced organ damage and hematological disturbances, coupled with restoring metabolic homeostasis and modulating gut microbiota. SFD has been found to possess specifically enriched beneficial bacterial genera, linked to the activation of brown adipose tissue and non-shivering thermogenesis. This study has provided initial evidence for a gut microbiota-metabolism axis mediating SFD's protective effect.

**Conclusion:**

SFD prolonged survival in rats with seawater-induced hypothermia, likely by enhancing thermogenesis and regulating lipid metabolism through gut microbiota changes. The findings highlighted the potential of SFD for hypothermia prevention; however, its exact underlying mechanisms require further validation.

## 1 INTRODUCTION

Accidental hypothermia, defined by a core body temperature falling below 35°C unintentionally, carries substantial risks of morbidity and mortality [1, 2]. Between 2000 and 2019, approximately 4.6 million people globally died each year due to cold-related conditions [3, 4]. This mortality burden has historically been exacerbated by conflicts and natural disasters, such as avalanches, earthquakes, and tsunamis [5]. Moreover, seawater immersion, a form of accidental hypothermia, poses a significant risk of rapid body temperature loss because seawater has a thermal conductivity approximately 24 times greater and a specific heat capacity 4 times higher than that of air [6], which accelerates the onset of hypothermia in individuals who fall overboard. Furthermore, immersion in seawater can elevate the risk of chronic wounds among coastal inhabitants and sailors, possibly resulting in life-threatening complications, such as hypercarbia, metabolic acidosis, severe electrolyte imbalances, and clinical deterioration [7, 8].

Clinically, hypothermia is categorized by core body temperature into mild (35°C to 32°C), moderate (32°C to 28°C), and severe (below 28°C) [9]. Its progression adversely affects multiple organs, leading to reduced cardiac contractility, arrhythmias, and impaired coagulation [10-12]. These changes are accompanied by declining vital signs, which significantly increase the risk of cardiac arrest [12]. Typically, passive and active external rewarming is required for most hypothermia patients, whereas more invasive internal techniques and extracorporeal life support with extracorporeal membrane oxygenation (ECMO) are reserved for those with refractory hypothermia or cardiac arrest [13]. However, managing hypothermia or rewarming-induced arrhythmias remains a challenge, as most medications are ineffective under hypothermic conditions [5, 14].

Traditional Chinese medicine, a prominent form of complementary and alternative therapy, has been valued for its efficacy in addressing critical conditions, like hypothermia, heatstroke, and burns [15, 16]. Shenfu decoction (SFD), a classic formula originating from the Ming Dynasty's Zheng Ti Lei Yao, boasts a 500-year clinical legacy. Comprising *Panax ginseng* C.A. Mey and *Aconitum carmichaelii* Debeaux, SFD has potent effects of Qi-invigorating and Yang-restoring, and is a wonder medication to treat Yang deficiency [17]. Clinically, SFD has demonstrated benefits in alleviating hypoxemia, reducing inflammation, managing septic shock, improving hemodynamics, protecting the cardiovascular system, and treating chronic heart failure [18, 19].

The vast and unpredictable nature of the marine environment makes maritime search and rescue particularly challenging. Prolonging survival time during seawater immersion is critical for improving victim outcomes in such emergencies. This study presents pioneering evidence that SFD may extend survival time in rats with hypothermia caused by seawater immersion. By integrating metabolomic and gut microbiota analyses, it offers novel insights into the mechanisms underlying SFD's effects on hypothermic scenarios. The overall experimental flow is shown in Fig. (**1**).

## 2 MATERIALS AND METHODS

### 2.1 SFD Preparation

Red ginseng (*Panax ginseng* C.A. Mey, batch no. 2021080007) and processed aconite lateral root (*Aconitum carmichaelii* Debeaux, batch no. 2008093) were purchased from Lei Yunshang Group Company (Shanghai, China). The mixture of these two herbs (1:1) was soaked in five volumes of water for 1 hour, and then decocted for 2 hours in a Chinese herbal decoction pot. This process was repeated three times. The obtained decoction was then concentrated to 1.08 g/mL and stored at -20°C [20].

### 2.2 Ultra-performance Liquid Chromatography Quadrupole Time-of-Flight Mass Spectrometry (UPLC-Q-TOF-MS) Analysis

Following a 5-minute centrifugation at 12,000 rpm, the supernatant from the SFD underwent filtration through a 0.22 μm organic phase filter membrane before analysis. A Waters H-Class UPLC system (Waters Corp., Beijing, China) was used for UPLC-Q-TOF-MS analysis, in conjunction with an AB Sciex Triple TOF^®^ 4600 mass (high-resolution mass spectrometry (HRMS); SCIEX, Shanghai, China). Chromatographic separation utilized a Waters CORTECS^®^ UPLC^®^ T3 column (2.1×100 mm, 1.6 µm), kept at a temperature of 30°C. The flow rate was set to 0.3 mL/min, and the injection volume was 2 µL. Acetonitrile (A, high-performance liquid chromatography (HPLC) grade, batch no. I1133829105, Merck, Germany) and 0.1% formic acid in water (B, HPLC grade, batch no. I139035113, Merck, Germany) formed the mobile phase. Gradient elution was performed with the following program: 0-5 minutes, 0% A; 5-10 minutes, 0–5% A; 10-20 minutes, 5-15% A; 20-30 minutes, 15-20% A; 30-35 minutes, 20-30% A; 35-38 minutes, 30% A; 38-48 minutes, 30-45% A; 48-55 minutes, 45-90% A; 55-58 minutes, 90% A; 58-58.1 minutes, 90-0% A; and 58.1-60 minutes, 0% A. MS employed electrospray ionization (ESI), operating in both positive and negative ion modes. The TOF mass range spanned m/z 50 to 1,700, with the ion source temperature maintained at 500°C. A spray voltage of +5000 V was applied in positive mode and -4500 V in negative mode. Both ion source gases 1 and 2 were adjusted to a pressure of 50 psi, with curtain gas at 35 psi. The settings included a declustering potential of 100 V and a collision energy of 10 eV. Data acquisition was performed using Analyst TF 1.7.1 software, and data processing was conducted with PeakView 1.2 software. Compound identification was performed by matching MS data with the natural products HR-MS/MS spectral library 1.0, screening compounds based on peak scores, and confirming identities using MS^1^ and MS^2^ spectral data.

### 2.3 Rat Model of Seawater-induced Hypothermia

Male Sprague-Dawley (SD) rats (220±10 g, specific pathogen-free) aged 6-8 weeks were purchased from Cyper-Bike Experimental Animal Co., Ltd. (Shanghai, China). Ethics approval was obtained from the Shanghai Changhai Hospital ethics committee (response V2 20250902, submitted on 2025-09-04). The rats were acclimated for a week under controlled conditions (temperature: 22±2°C, relative humidity: 50±10%, 12-hour light/dark cycle) with ad libitum access to standard chow and water. Animals underwent daily health monitoring (including activity, food intake, and body temperature) at fixed times.

The rat model of seawater-induced hypothermia was established using a platform that was independently designed by our team and has been granted two national utility model patents in China (CN218606938U, CN218899782U). The platform featured an upright rat restraint container, a seawater tank, and an automatic thermostatic controller (Supplementary Fig. **S1**). It ensured rapid seawater circulation and maintained a stable temperature of 15°C for inducing hypothermia in conscious, restrained rats.

### 2.4 Survival Analysis

To ascertain the optimal SFD dosage, a total of 60 rats were randomly assigned to six groups by computer-generated random numbers, with 10 rats allocated to each group. Rats in the control group were administered distilled water, whereas rats in five SFD groups were treated with five different concentrations of SFD. The dosage of SFD started from 0.675 g/kg and increased sequentially by doubling up to 10.8 g/kg. Following a single gavage, all rats were immediately immersed in 15°C simulated seawater. Electrocardiograph signals of each seawater-induced hypothermia rat were monitored. The death of a rat was recorded by observing an irreversible flat line on the electrocardiograph. During the whole process, rats were under continuous observation. Any adverse events (*e.g.*, accidental drowning, water aspiration) that led to non-experimental death triggered immediate termination of the rats in accordance with humane endpoint guidelines.

### 2.5 Blood Collection and Analysis

To further investigate the impact of SFD on hypothermia induced by seawater immersion in rats, 36 rats were randomly organized into three experimental groups (control, seawater, and SFD; n=12). The control and seawater groups were administered with distilled water, while the SFD group received the optimal SFD dose (1.35g/kg) based on the results of survival analysis. Following a single gavage, rats in the seawater and SFD groups were immersed in seawater at 15°C for 2 hours, while rats in the control group were kept at room temperature. Sodium pentobarbital was used to anesthetize all rats, and blood samples were collected from the abdominal aorta for further analysis. Consistent with the survival time analysis experiment, the investigators also paid close attention to the general condition of the rats.

#### 2.5.1 Hematological Analysis

Hematological parameters, including red blood cell count (RBC), hemoglobin (HGB), hematocrit (HCT), and platelet count (PLT), were determined using a Siemens complete blood count kit (45344; Siemens AG, Germany).

#### 2.5.2 Biochemical Analysis

Biochemical markers, such as glucose (GLU), calcium (Ca), phosphorus (P), blood urea nitrogen (BUN), alkaline phosphatase (ALP), total protein (TP), and albumin (ALB), were analyzed using reagent kits (220822101, 231214201, 220221101, 221014201, 220424301, 220620101, and 220226301, respectively; Meikang Biotechnology Co., Ltd., Ningbo, China) with a HITACHI-7080 automated biochemical analyzer (Hitachi High-Tech Corp., Tokyo, Japan).

#### 2.5.3 Inflammatory and Cardiac Biomarker Analysis

The quantification of inflammatory cytokines, including interleukin-1 beta (IL-1β), interleukin-6 (IL-6), and tumor necrosis factor-alpha (TNF-α), was conducted with enzyme-linked immunosorbent assay (ELISA) kits (A301B21114, A38221144, and A31021224, respectively; MultiSciences Biotech Co., Ltd., Hangzhou, China). Cardiac troponin T (cTnT) was measured using an ELISA kit (BPE30600; Langton Biotechnology Co., Ltd., Shanghai, China).

### 2.6 Metabolomics Analysis

The same animal grouping and intervention protocols as in the blood analysis study were applied. After orbital sinus blood collection, abdominal aortic blood was obtained and centrifuged. Serum was stored at −80°C. Following metabolomics quality control, 10 samples in each group were randomly selected using computer-generated numbers for analysis.

This study utilized an advanced MS platform comprising the Waters ACQUITY UPLC I-Class Plus (Waters Corp., Milford, MA, USA) in conjunction with the Thermo QE HF high-resolution mass spectrometer (Thermo Fisher Scientific, Waltham, MA, USA), and the Agilent 7890B-5977B (Agilent Technologies Corp., Santa Clara, CA, USA) gas chromatography-mass spectrometry (GC-MS) system. Data processing and metabolite identification workflows were implemented using specialized software tools, as outlined below.

Liquid chromatography-mass spectrometry (LC-MS): Separation was performed on an ACQUITY UPLC HSS T3 column (100 mm×2.1 mm, 1.8 µm) at 45°C, with a flow rate of 0.35 mL/min and an injection volume of 3 µL. The mobile phase consisted of water containing 0.1% formic acid (A117-50, Fisher Scientific, HPLC, 99.0%) and acetonitrile (ACN, A998-4, Fisher Scientific, HPLC, 99.95%). ESI was used in positive and negative ion scanning modes with data-dependent acquisition. Raw LC-MS data were preprocessed using Progenesis QI v3.0 (Nonlinear Dynamics, Newcastle, UK) [21], a dedicated metabolomics software that facilitated baseline filtering, peak detection, peak integration, retention time alignment, and normalization. Key parameters for data processing included precursor ion tolerance: 5 ppm (Human Metabolome Database (HMDB) + LipidMaps)/10 ppm (LuMet-Animal + METLIN); product ion tolerance: 10 ppm (HMDB + LipidMaps)/20 ppm (LuMet-Animal + METLIN).

GC-MS: A DB-5MS capillary column (30 m×0.25 mm×0.25 μm, Agilent J&W Scientific, Folsom, CA, USA) was used for separation, with helium (99.999% purity) flow at 1.0 mL/min. The injector was set at 260°C, and a 1 μL sample was injected in splitless mode. Detection was performed using electron ionization at 230°C, with the quadrupole at 150°C and an electron energy of 70 eV, operating in comprehensive scan mode (m/z 50 to 500). Raw GC-MS data (.D format) were converted to .abf files using Analysis Base File Converter, then preprocessed in MS-DIAL v4.24 [22] *via* peak deconvolution, noise removal, and metabolite annotation (matching retention times, fragment ions, and isotope patterns against in-house/public databases). MS-DIAL further supported peak alignment, filtering, and missing value imputation to generate a normalized matrix with sample metadata, metabolite names, retention times, m/z values, and peak intensities.

Metabolite identification was performed using multiple dimensions (retention time, exact mass, fragment ions, and isotope distribution) with the following databases: The HMDB, LipidMaps (v2.3), METLIN, the local LuMet-Animal database (Luming Biology, Shanghai, China), and the LUG database (Untarget database of GC-MS from Lumingbio, Shanghai, China). For GC-MS experiments involving headspace sampling of volatile compounds and wax component detection, the National Institute of Standards and Technology database was used for qualitative and quantitative analyses.

### 2.7 Gut Microbiota Analysis

For this part of the study, the animal grouping and sample size were consistent with the blood analysis study. Following blood collection from the abdominal aorta, intestinal fecal samples were aseptically collected and stored at -80°C. The Fast Pure Feces DNA isolation kit (Majorbio Bio-pharm Technology Co., Ltd., Shanghai, China) was used to extract microbial DNA from fecal samples, which was quantified by a NanoDrop^®^ ND-2000 spectrophotometer (Thermo Scientific, USA). Primers 338F (5'-ACTCCTACGGGAGGCAG-CAG-3') and 806R (5'-GGACTACHVGGGTWTCTAAT-3') amplified the V3-V4 regions of the bacterial 16S rRNA gene on an ABI GeneAmp^®^ 9700 PCR system (ABI, USA) in accordance with standard PCR protocols. The purification of PCR products was carried out with the AxyPrep DNA gel extraction kit (Axygen Biosciences, Union City, CA, USA), followed by quantification using a Quantus™ fluorometer (Promega, USA). Sequencing was performed on a NextSeq 2000 platform (PE300 kit), and the data were submitted to the National Center for Biotechnology Information Sequence Read Archive (NCBI SRA, accession: PRJNA1083888). Raw reads were quality-checked using a fast all-in-one FASTQ preprocessor (fastp, v0.19.6) [23] and then combined with Fast Length Adjustment of Short reads (FLASH, v1.2.11) [24, 25]. The clustering of operational taxonomic units (OTUs) at 97% similarity was performed using UPARSE (v11) [26, 27]. Additionally, the Ribosomal Database Project Classifier (v2.13) [28, 29] was used to determine taxonomy using the Silva 16S rRNA database.

### 2.8 Statistical Analysis

Statistical analysis was performed using SPSS 21.0. Quantitative data were expressed as mean ± SD. For survival analysis, Kaplan-Meier curves estimated rat survival rates, with group differences evaluated by the log-rank test. For blood sample analysis, differences between two groups were assessed using the Student's t-test for normally distributed data with equal variances, or the Mann-Whitney U test for non-normally distributed data or unequal variances. Significance was set at *p*<0.05.

GC-MS and LC-MS/MS data were processed for peak detection and normalized, using internal standards (relative standard deviation<0.1) for quality control. Group differences in compound intensities were assessed *via* the Wilcoxon rank-sum test. Statistical analyses were conducted using R software, with partial least squares discriminant analysis (PLS-DA) applied to identify group-specific metabolites. Cross-validation and permutation tests ensured model reliability. Metabolites with variable importance in projection>1.0 and *p*<0.05 were deemed significant.

The Majorbio Cloud Platform was used to analyze gut microbiota data. Alpha diversity indices (Chao1, Shannon) were computed using mothur (version 1.30.2) [30], with group differences evaluated by the Kruskal-Wallis test. Principal coordinates analysis (PCoA) with Bray-Curtis distances assessed microbial community similarities, with permutational multivariate analysis of variance testing group differences. Specific microbiota differences were identified through the Wilcoxon rank-sum test. Significant taxonomic differences at the genus level were determined using linear discriminant analysis (LDA) effect size (LDA>3, P<0.05) [31]. Correlation network analysis was conducted using Spearman correlation (|r|>0.6, *p*<0.05) [32].

## 3 RESULTS

### 3.1 SFD Composition

**Fig. (2):**
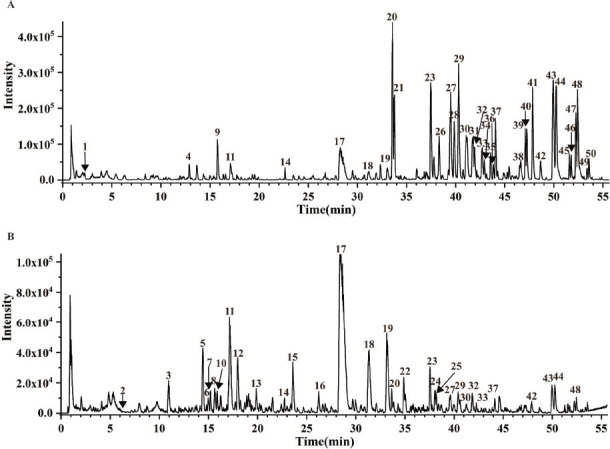
UPLC-Q-TOF/MS analysis of chemical composition in SFD. Intensity of ion flow of SFD at different retention times in negative (**A**) and positive (**B**) ion modes.

UPLC-Q-TOF/MS analysis of the SFD extract identified a total of 50 compounds (Fig. **2**, Supplementary Table **S1**), as determined by multistage MS data and comparison with a high-resolution natural products database and relevant literature [33-40]. The identified constituents comprised a chemically diverse mixture, including triterpenoid saponins (*e.g.*, ginsenosides Rg1, Rf, Rh1), phenolic acids (*e.g.*, eucomic acid), and alkaloids (*e.g.*, benzoylmesaconine), reflecting the complex and multi-herb nature of the traditional formula. Notably, compounds, such as ginsenosides and phenolic components, which have been previously reported to exhibit cardioprotective and anti-inflammatory activities [41, 42], may lay an important material foundation for the pharmacological effects of SFD under hypothermic conditions.

### 3.2 The Effect of SFD on Survival Times in Seawater-immersed Hypothermic Rats

**Fig. (3):**
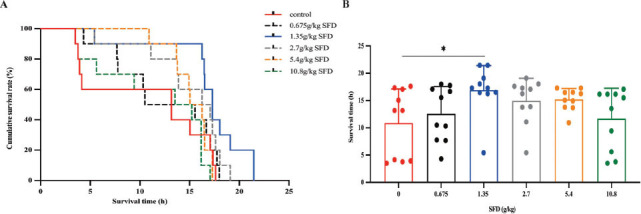
Effect of SFD on survival time in seawater-immersed hypothermia rats. (**A**) Survival curves; (**B**) mean survival times. n=10 per group, *p<0.05.

Survival analysis revealed that hypothermic seawater immersion posed a significant lethal threat to rats, with a mean survival time of 10.88 ± 6.25 hours observed in the control group (Figs. **3A** and **3B**). Pretreatment with SFD at doses ranging from 0.675 to 10.8 g/kg prior to immersion resulted in mean survival times ranging from 11.70 ± 5.57 hours to 16.94 ± 4.48 hours (Fig. **3B**). Notably, the prolongation of survival time in seawater immersion-induced hypothermia did not exhibit a dose-dependent relationship with SFD concentration. Statistical analysis further indicated that only pretreatment with 1.35 g/kg SFD significantly extended the mean survival time by approximately 6 hours compared to the control group (*p*=0.015).

### 3.3 The Effect of SFD on Hematological and Biochemical Changes in Seawater-immersed Hypothermic Rats

#### 3.3.1 Hematological Analysis Results

**Fig. (4):**
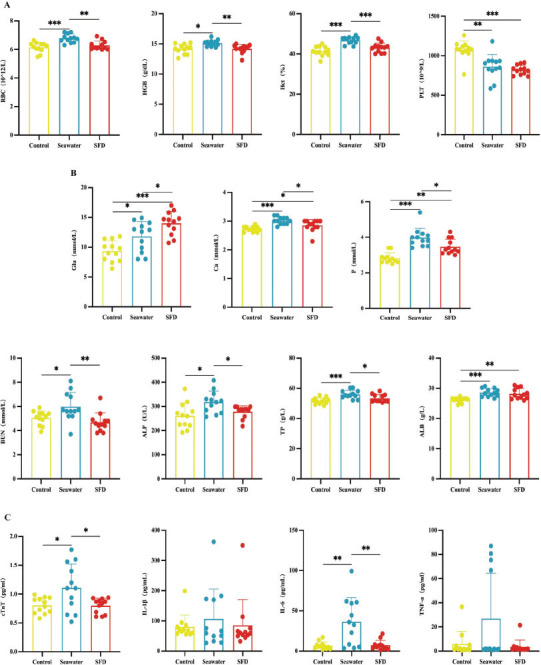
Effect of SFD on hematological and biochemical changes in seawater-immersed hypothermic rats. (**A**) Hematological parameters (RBC, HGB, Hct, PLT); (**B**) biochemical parameters (GLU, Ca, P, BUN, ALP, TP, ALB); (**C**) cardiac injury markers (cTnT) and inflammatory cytokines (IL-6, IL-1β, TNF-α). n=12 per group, *p<0.05, **p<0.01, ***p<0.001.

Seawater immersion induced significant hematological alterations in rats (Fig. **4A**), characterized by marked elevations in RBC, HGB, and Hct (all *p*<0.01), consistent with hemoconcentration [43, 44]. In contrast, PLT was significantly reduced from 1074.50±114.72×10^9^/L to 862.17±155.07×10^9^/L (*p*=0.003), implying possible microvascular involvement. The SFD group displayed a divergent profile: while PLT remained lower than that in the control group (*p*=0.000), RBC, HGB, and Hct were all significantly lower than in the seawater group (all *p*<0.01). These results indicated SFD to elicit a hematological response distinct from that of seawater immersion alone.

#### 3.3.2 Biochemical Analysis Results

As shown in Fig. (**4B**), seawater immersion hypothermia induced significantly elevated several biochemical parameters in rats, including GLU, Ca, P, BUN, ALP, TP, and ALB. This increase may be attributed to hemoconcentration caused by hyperosmotic seawater [43, 44], along with organ dysfunction and electrolyte disturbances resulting from low-temperature seawater exposure. Pretreatment with SFD by gavage normalized BUN, ALP, and TP levels, and reduced serum Ca and P levels compared to the seawater immersion group, but it had no significant effect on ALB. Notably, while low-temperature seawater immersion increased GLU from 9.34±1.75 mmol/L to 11.80±2.51 mmol/L (*p*=0.011), SFD administration further elevated GLU to 14.01±1.96 (*p*=0.000 *vs*. control, *p*=0.025 *vs*. seawater), suggesting that SFD may modulate energy and substrate metabolism.

#### 3.3.3 Inflammatory and Cardiac Biomarker Results

As illustrated in Fig. (**4C**), the cardiac injury marker cTnT was significantly elevated from 0.81±0.14 pg/mL to 1.11±0.41 pg/mL (*p*=0.031), indicating myocardial stress caused by low-temperature seawater immersion. SFD treatment exhibited a significant reduction in cTnT levels (0.80±0.13 pg/mL, P=0.046 vs. seawater group), approaching control values, suggesting potential mitigation of cold seawater-induced cardiac injury by SFD. The pro-inflammatory cytokine IL-6 remained at 6.68±4.53 pg/mL in the control group, but increased to 36.46±30.00 pg/mL (*p*=0.008). However, IL-6 concentrations of rats were markedly lower in the SFD group compared to the seawater group (7.2±6.09, *p*=0.007), reflecting SFD’s potential to suppress seawater-triggered inflammation. No significant differences were observed in IL-1β or TNF-α levels across the groups.

### 3.4 Metabolomics Analysis of SFD Administration in Seawater-immersed Hypothermic Rats

**Fig. (5):**
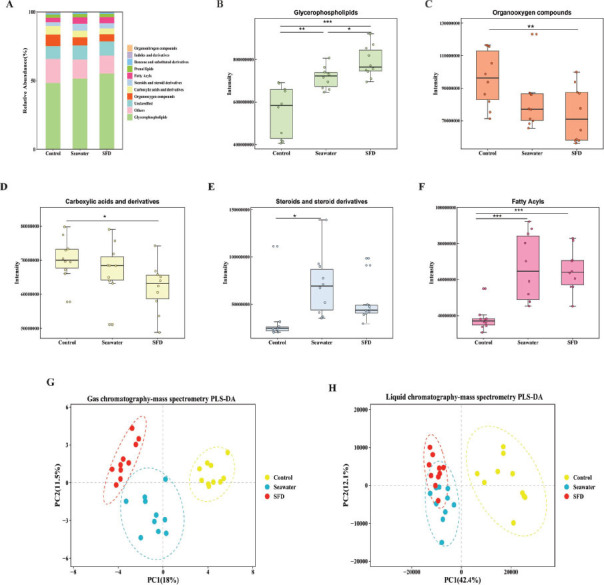
Metabolic profile and multivariate data assessment from GC-MS and LC-MS. (**A**) Metabolite distribution across groups. Intensity of five key metabolites, including glycerophospholipids (**B**), organooxygen compounds (**C**), carboxylic acids and derivatives (**D**), steroids and steroid derivatives (**E**), fatty acyls (**F**). PLS-DA analysis of GC-MS (**G**) and LC-MS (**H**). n=10 per group, *p<0.05, **p<0.01, ***p<0.001.

The analysis of serum metabolites was conducted using LC-MS and GC-MS across both ion modes, identifying nine preliminary categories (Fig. **5A**). Consistent with the results of serum GLU analysis, the intensity of glycerophospholipids was elevated by seawater immersion, and SFD even raised it to a higher level (Fig. **5B**). SFD administration triggered remarkable intensity drop of organooxygen compounds (Fig. **5C**), and carboxylic acids and derivatives (Fig. **5D**), whereas a significantly different intensity of the above two categories was observed in the seawater group. Seawater immersion caused the up-regulation of fatty acyls, as well as steroids and steroid derivatives; however, SFD administration only elevated the intensity of fatty acyls (Fig.**5E**, Fig.**5F**). PLS-DA demonstrated distinct separation of metabolic profiles between the groups (Fig. **5G**, Fig. **5H**).

**Fig. (6):**
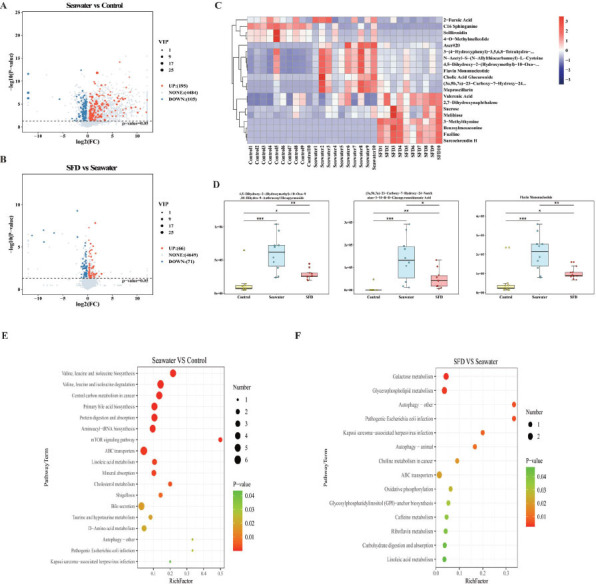
Differential metabolite classification and pathway analysis. Upregulated and downregulated metabolites in seawater *vs*. the control group (**A**) and SFD vs. seawater group (**B**). (**C**) 20 significantly altered metabolites between seawater and SFD groups. (**D**) Intensity of three potential biomarkers modulated by SFD in seawater-immersed hypothermia. KEGG pathway enrichment analysis for differential metabolites in seawater *vs*. control group (**E**) and SFD *vs*. seawater group (**F**). n=10 per group, *p<0.05, **p<0.01, ***p<0.001.

Volcano plots revealed 195 upregulated and 105 downregulated metabolites in the seawater (Fig. **6A**). Moreover, when compared to rats in the seawater immersion group, 66 upregulated and 71 downregulated metabolites were identified in the SFD-administered rats (Fig. **6B**), along with 20 potential biomarkers visualized *via* a cluster heatmap (Fig. **6C**). Among these, valerenic acid, 2,7-dihydroxynaphthalene, sucrose, melibiose, 3-methylthymine, benzoylmesaconine, fuziline, and sarcoehrendin H were significantly elevated, while others were reduced. Notably, 4,5-dihydroxy-2-(hydroxymethyl)-10-oxo-9,10-dihydro-9-anthracenyl hexopyranoside, (3a,5b, 7a)-23-carboxy-7-hydroxy-24-norcholan-3-yl-β-D-glucopyranosiduronic acid, and flavin mononucleotides were markedly elevated by low-temperature seawater immersion, but were strongly decreased by SFD administration, indicating their potential as biomarkers (Fig. **6D**).

The Kyoto Encyclopedia of Genes and Genomes (KEGG) pathway enrichment analysis showed that the differential metabolites between the seawater and control groups were primarily associated with pathways, such as valine, leucine, and isoleucine biosynthesis and degradation, primary bile acid biosynthesis, protein digestion and absorption, aminoacyl-tRNA biosynthesis, mTOR signaling, ABC transporters, and linoleic acid metabolism (Fig. **6E**). For the SFD vs. seawater comparison, significant enrichment was observed in pathways related to galactose metabolism, glycerophospholipid metabolism, and ABC transporters (Fig. **6F**). Notably, lipid metabolism, particularly glycerophospholipid and linoleic acid metabolism, emerged as a key pathway relevant to seawater-immersed hypothermia and its modulation by SFD.

### 3.5 Gut Microbiota Analysis of SFD Administration in Seawater-immersed Hypothermic Rats

**Fig. (7):**
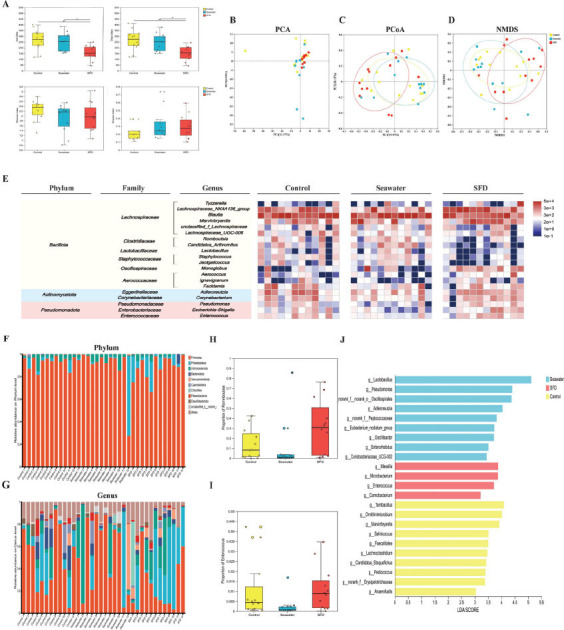
Analysis of gut microbiota diversity and composition. (**A**) α-diversity differences were evaluated by 4 indices, including Ace, Chao, Shannon, and Simpson. β-diversity differences among microbiota were shown by PCA (**B**), PCoA (**C**), and NMDS (**D**). (**E**) Heatmap of microbial composition (genus, family, phylum levels). Phylum (**F**) and genus (**G**) level composition among rats. Relative abundances of key genera *Enterococcus* (**H**) and *Romnboutsia* (**I**). (**J**) Taxa with significant differences (LDA score>3) are shown. n=12 per group, *p<0.05, **p<0.01.

The abundance-based coverage estimator (ACE) index, reflecting gut microbiota richness, revealed that the SFD treatment significantly reduced colony richness (Fig. **7A**) [45]. This finding indicated that SFD downregulated gut microbiota richness based on OTU distribution, while the diversity remained unaffected. To assess microbial community composition and inter-group differences, principal component analysis (PCA; Fig. **7B**), PCoA (Fig. **7C**), and non-metric multidimensional scaling (NMDS; Fig. **7D**) were performed [46]. Both PCoA and NMDS analyses indicated significant variations in β-diversity across the three groups (*P*<0.05).

A heatmap illustrating microbial composition at the genus, family, and phylum levels (Fig. **7E**) further clarified genetic relationships and relative abundance. The species composition (Fig. **7F**, Fig. **7G**) indicated Firmicutes, Proteobacteria, and Actinobacteriota to be dominant at the phylum level, while *Lactobacillus*, *Romboutsia*, and *Staphylococcus* were prevalent at the genus level. The Wilcoxon rank-sum test identified notable differences in the relative abundance of 10 gut microbial genera comparing the control group with the seawater group, as well as 15 genera when contrasting the seawater group with the SFD group. Among these, *Romboutsia* and *Enterococcus* were identified as representative genera (Fig. **7H**, Fig. **7I**). Their proportions in the SFD group were approximately 3 times and 4.3 times higher, respectively, than those in the seawater group, while showing negligible differences compared to the control group.

LDA further identified 17 genera with LDA scores>3 as key contributors to differences among the groups (Fig. **7J**) [47]. Dominant genera in the SFD group included *Massilia*, *Microbacterium*, *Enterococcus*, and *Carnobacterium*. These results demonstrated SFD to modulate key microbial populations, contributing to a rebalanced gut microbiota composition.

### 3.6 Correlating Analysis of SFD Administration in Seawater-immersed Hypothermic Rats

**Fig. (8):**
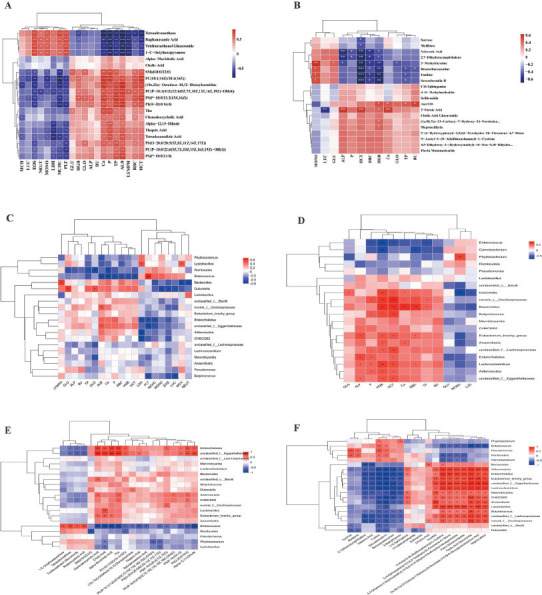
Correlation heatmap among blood parameters, metabolites, and gut microbiota. Spearman’s rank correlation heatmaps between blood parameters and differential metabolites in the seawater vs. control (**A**) and SFD *vs*. seawater groups (**B**). Correlations between blood parameters and differential gut microbiota in the seawater *vs*. control (**C**) and SFD *vs*. seawater groups (**D**). Correlations between differential metabolites and gut microbiota in the seawater *vs*. control (**E**) and SFD *vs*. seawater groups (**F**).

To explore the relationships among biochemical indexes, gut microbiota, and metabolites, Spearman’s rank correlation-based heatmaps were generated. Key variables were identified based on significant differences (*p*<0.05) in seawater vs. control and SFD *vs*. seawater comparisons, focusing on the top 20 gut microbiota and metabolites (Fig. **8**). Results revealed strong associations between biochemical indexes and metabolites during seawater-immersed hypothermia. For instance, *Enterococcus* was negatively correlated with ALB, Ca, P, and metabolites, such as chenodeoxycholic acid, cholic acid, alpha-muricholic acid, and others. In the SFD vs. seawater comparison, HCT, RBC, and HGB levels showed negative correlations with metabolites, like valerenic acid and benzoylmesaconine, while demonstrating positive associations with gut microbiota, such as norank_f_Oscillospiraceae, Bacteroides, and Eubacterium_brachy_group. Notably, benzoylmesaconine and fuziline, compounds from *Aconitum carmichaelii* Debeaux, were strongly negatively correlated with RBC, HGB, and HCT levels, as well as microbiota, including Adlercreutzia, Enterorhabdus, and Lachnoclostridium.

## 4 DISCUSSION

The primary approach to managing seawater-immersed hypothermia involves minimizing heat loss and initiating rewarming promptly. However, rewarming is often impractical in emergency settings due to limited access to equipment and restricted transport time within 1 hour [48, 49]. The Hypothermia Prevention and Management Kit by North American Rescue, designed for active rewarming, shows limitations in maintaining thermal balance in cold conditions due to inadequate insulation [50, 51]. Hypothermia, a critical condition affecting multiple organ systems, lacks effective prevention strategies [5, 14]. Given the established use of SFD and its derivatives (*e.g.*, SF injection) in treating conditions, such as heart failure, acute myocardial infarction, and shock [52-54], we hypothesized that SFD could have therapeutic potential for seawater-immersed hypothermia and conducted experiments to explore this possibility.

The chemical analysis of SFD revealed a diverse composition, with triterpenoid saponins, phenolic acids, and alkaloids representing key bioactive classes. These constituents, particularly ginsenosides and phenolic compounds, aligned with established cardioprotective and anti-inflammatory properties, providing a mechanistic basis for the efficacy of SFD under hypothermic stress [55-58]. Such structural features underscore a rational foundation for mitigating hypothermia-induced injuries, including myocardial damage, inflammation, and metabolic dysfunction. Further research is needed to clarify the bioavailability, pharmacokinetics, and target-specific interactions of these compounds in hypothermia-exposed models, which is essential for identifying the key active components driving the protective effects of SFD.

Under cold seawater exposure, survival time is a critical indicator of physiological resilience. Significant differences in survival time and mortality were observed among rats under varying cold seawater conditions [59]. In this study, the 1.35 g/kg SFD group significantly prolonged survival time and improved survival rates, underscoring the protective effects of SFD against hypothermia induced by seawater immersion. Prolonged immersion in cold seawater leads to severe hematological abnormalities. Hypothermia disrupts coagulation by increasing blood viscosity, impairing platelet function, and inhibiting clotting cascades, potentially causing microinfarctions [60]. Immersion elevated RBC count and HCT levels while reducing PLT count; SFD treatment reversed most of these changes, except for PLT. The elevated levels of cTnT were alleviated by SFD, indicating its promising preventive effect against cardiac injury. Previous documents have shown that hypothermia can impair kidney function through tubular dysfunction, renal vasoconstriction, and toxin retention, leading to reduced clearance of toxic metabolites and metabolic acidosis [61]. It can also promote systemic inflammation by increasing pro-inflammatory cytokines, such as IL-6, IL-1β, and TNF-α [42]. After seawater immersion, significantly elevated IL-6 and TP levels, along with increased but not statistically significant IL-1β and TNF-α levels, suggested potential intestinal bacterial translocation and cytokine cascade activation [62]. Our results indicated SFD to significantly decrease IL-6 levels, implying its survival-prolonging effect to be mediated by mitigating inflammation.

Metabolomics, complementing other 'omics' fields, offers insights into metabolic states and biomarkers for diseases, with metabolites playing crucial roles in disease mechanisms [63]. This study used untargeted metabolomics *via* LC-MS and GC-MS to examine metabolic changes from low-temperature seawater immersion and SFD treatment. SFD notably normalized altered metabolites, like flavin mononucleotide (FMN), cholic acid glucuronide, and meproscillarin [64]. FMN, vital for enzymatic processes, is key in the mitochondrial electron transport chain and redox homeostasis [65, 66]. Seawater-induced hypothermia elevates FMN levels due to mitochondrial complex I (C-I) dysfunction, disrupting cellular energy metabolism [67]. SFD appears to alleviate these disturbances by restoring C-I activity and stabilizing FMN levels. Consequently, the proton motive force is re-established, and adenosine triphosphate synthesis is promoted. KEGG analysis of riboflavin metabolism suggests that this recovery may also influence other metabolic pathways. These observations suggest that elevated FMN levels under hypothermic stress might result from reversible FMN release from C-I, impairing enzyme activity and redox balance. SFD likely mitigates these effects by stabilizing mitochondrial function. However, the detailed molecular pathways and causal links remain speculative and require further experimental validation. In addition, KEGG analysis highlighted glycerophospholipid metabolism, with phosphatidylcholine and phosphatidylethanolamine (PE) identified as predominant metabolites. PE, a critical component of cell membranes, plays a vital role in energy metabolism and thermoregulation. Notably, PE modulates thermogenesis through uncoupling protein 1 (UCP1) activity in brown and beige adipocytes [68, 69]. Cold exposure increases PE levels, but mitochondrial PE deficiencies correlate with cold intolerance, emphasizing PE's role in energy homeostasis under thermal stress [70].

Gut microbiota perform essential functions unachievable by the host, influencing metabolism and health *via* metabolites serving as substrates or signaling molecules [71]. They are associated with various diseases, including hypothermia. Studies reveal that cold acclimation reshapes gut microbiota, enhancing host metabolism [72]. This study marks the first metabolomics and gut microbiota analysis in seawater-immersed hypothermia. In terms of alpha diversity, microbial richness and diversity in seawater-immersed rats decreased compared to controls, but unexpectedly increased relative to the SFD group. We hypothesized SFD combined with seawater immersion to provide an added stimulus, causing transient microbial disturbance and hindering equilibrium restoration. This disturbance may benefit host recovery *via* specific microbial compositions. At the phylum level, the abundance of Firmicutes in the SFD group decreased compared to the seawater group, whereas Proteobacteria exhibited an opposite trend. Research indicates that intestinal Firmicutes are negatively correlated with body temperature, while Proteobacteria show a positive correlation across diverse hosts and conditions, including temperature stress. This suggests that SFD might aid in recovering core temperature during seawater-immersed hypothermia. At the genus level, *Enterococcus* abundance declined in the seawater group compared to controls but significantly increased in the SFD group, where it was identified as a key microbe. *Enterococcus* has been linked to brown adipose tissue (BAT) activation. For example, *P. ginseng*-induced *Enterococcus* produces long-chain fatty acids, like myristoleic acid, to stimulate BAT activation [73], while ginsenoside Rg1-induced *Enterococcus* upregulates UCP1 expression, reducing lipid droplet size and hepatic lipid accumulation [62, 74]. Similarly, *Romboutsia* abundance increased in the SFD group and has been associated with treatments, like Heimao tea and gamma-aminobutyric acid, which promote thermogenic gene expression (*e.g*., Ucp1, Prdm16, Pgc1α) in adipose tissue [75, 76]. Other microbes, such as *Lachnospiraceae* NK4A136 and *Candidatus Arthromitus*, also enhance non-shivering thermogenesis [77, 78].

## STUDY LIMITATIONS

This study involved two primary limitations. First, while the rat model of seawater immersion-induced hypothermia is commonly utilized for exploring prevention and resuscitation strategies, the differences in thermoregulation between species restrict its capacity to accurately reflect human pathophysiology. Second, although metabolomic and gut microbiota analyses indicate potential links between SFD and extended survival, the mechanistic validation, which includes proteomic profiling of thermogenic pathways and functional microbiota transplantation, requires further investigation. Future targeted molecular studies are essential to address these gaps.

## CONCLUSION

In summary, our findings proved that SFD remarkably prolonged the survival time of seawater-immersed hypothermia rats, and this can mainly be attributed to the thermogenesis-promoting effect of SFD, which could be a result of lipid metabolism regulation and thermogenesis-related gut microbiota. Our endeavor provided a novel insight into the effects of the traditional Chinese medicinal decoction for seawater-immersed hypothermia. These findings indicate that SFD could be further advanced into a promising alternative drug therapy for seawater-immersed hypothermia.

## Figures and Tables

**Fig. (1):**
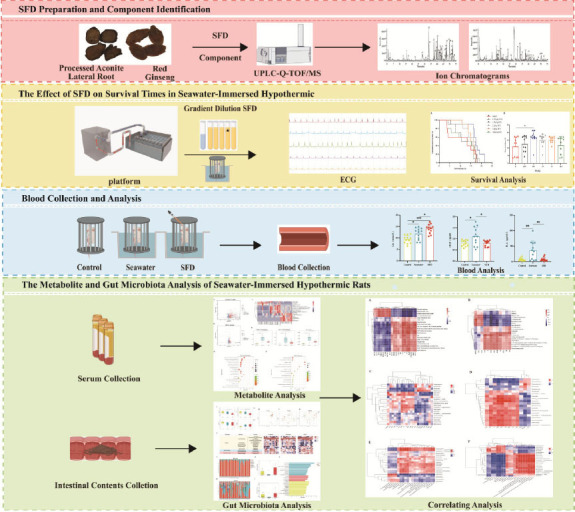
Workflow of SFD’s effects in seawater-immersed hypothermia rats.

## Data Availability

The data and supportive information are available within the article.

## LIST OF ABBREVIATIONS

ACE: Abundance-based Coverage Estimator
ALB: Albumin
ALP: Alkaline Phosphatase
BAT: Brown Adipose Tissue
BUN: Blood Urea Nitrogen
Ca: Calcium
cTnT: Cardiac Troponin T
C-I: Complex I
ECG: Electrocardiogram
ECMO: Extracorporeal Membrane Oxygenation
ELISA: Enzyme-linked Immunosorbent Assay
ESI: Electrospray Ionization
FLASH: Fast Length Adjustment of Short Reads
FMN: Flavin mononucleotide
GC-MS: Gas Chromatography-mass Spectrometry
GLU: Glucose
HCT: Hematocrit
HGB: Hemoglobin
HMDB: Human Metabolome Database
HPLC: High-performance Liquid Chromatography
IL-1β: Interleukin-1 Beta
IL-6: Interleukin-6
KEGG: Kyoto Encyclopedia of Genes and Genomes
OTUs: Operational Taxonomic Units
LC-MS: Liquid Chromatography-mass Spectrometry
LDA: Linear Discriminant Analysis
MS: Mass Spectrometry
NCBI SRA: National Center for Biotechnology Information Sequence Read Archive
NMDS: Non-metric multidimensional scaling
PCoA: Principal Coordinates Analysis
PCA: Principal Component Analysis
PE: Phosphatidylethanolamine
PLS-DA: Partial Least Squares Discriminant Analysis
PLT: Platelet Count
RBC: Red Blood Cell Count
SFD: Shenfu Decoction
SD: Sprague-Dawley
TNF-α: Tumor Necrosis Factor-alpha
UCP1: Uncoupling Protein 1
