# Supplementary material for: Shenfu Decoction Extends Survival Time of Seawater-Induced Hypothermia in Rats: The Role of Metabolomics and Gut Microbiota
Source: Curr Drug Metab. 2026 Jan 20;27(1):58–74. doi: 10.2174/0113892002403722251122095723 (PMC13312379; doi:10.2174/0113892002403722251122095723)
Supplement: Supplementary file 1 [file CDM-27-1-58_SD1.pdf]

Supplementary Material

Shenfu Decoction Extends Survival Time of Seawater-Induced Hypothermia in Rats: The Role of Metabolomics and Gut Microbiota

Ya-jing Wang[1,2,#], Hong-zhi Chen[1,3,#], Zhi-bo Wang[1,3,#], Chao-yue Sun[1,3], Chen-yang Guo[1,3], Yi Ruan[1,4], Chuan-tao Li[5], Bin Zou[6,7], Zi-fei Yin[1,*] and Wei Gu[1,8,*]

[1]School of Traditional Chinese Medicine, Naval Medical University, Shanghai 200433, China; [2]Yueyang Hospital of Integrated Traditional Chinese and Western Medicine, Shanghai University of Traditional Chinese Medicine, Shanghai, 200433, China; [3]Basic Medicine College, Naval Medical University, Shanghai, 200433, China; [4]Department of Traditional Chinese Medicine, PLA Naval Medical Center, Shanghai, 200052, China; [5]Naval Medical Center of Naval Medical University Aviation Physiological and Psychological Training Forces, Shanghai, 200433, China; [6]Dujiangyan Air Force Special Service Sanatorium, Chengdu, 611838, China; [7]Department of Biochemistry and Molecular Biology, College of Basic Medical, Naval Medical University, Shanghai, 200433, China; [8]Department of Rehabilitation, Changhai Hospital, Naval Medical University, Shanghai, 200433, China

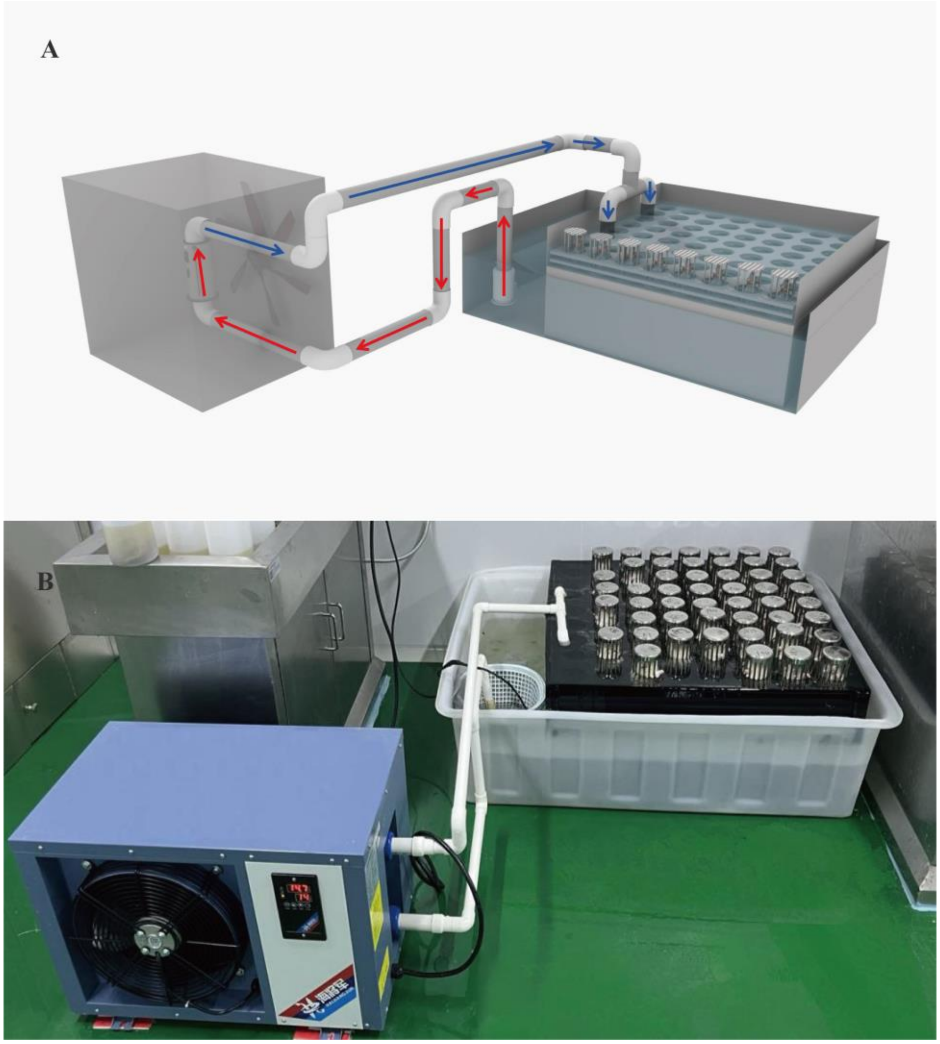

Fig. (S1A-B). The hypothermia platform for seawater immersion in rats included an upright rat restraint container, a seawater immersion sink, and an automatic temperature controller. (**A**) Schematic diagram of the platform. (**B**) Photograph of the physical setup of the platform.

Table S1. The main predicted chemical constituents of SFD and MS/MS data.

Number	Time (min)	Adduct Ion	Molecular Formula	Molecular Weight	English Name	MS/MS (m/z)	Origin	Compound
1	2.24	$[M-H]^-$	$C_9H_{12}N_2O_6$	244.07	Uridine	243.0615; 200.0554; 152.0350; 122.0250; 110.0251; 82.0209	Ginseng, Aconite	Nucleosides
2	5.96	$[M+H]^+$	$C_{10}H_{13}N_5O_5$	283.09	Guanosine	284.1427; 152.0565; 135.0298; 110.0345	Ginseng, Aconite	Nucleosides
3	10.96	$[M+H]^+$	$C_{22}H_{35}NO_5$	393.25	Karacolidine	394.2592; 376.2471; 358.2383; 340.2218	Aconite	Alkaloids
4	12.89	$[M-H]^-$	$C_{18}H_{24}O_{13}$	448.12	5-[(2-O-D-apio-β-D-furanosyl-β-D-glucopyranosyl)oxy]-2-hydroxybenzoic acid	447.1145; 315.0737; 207.0308; 152.0121; 108.0220	/	Phenolic acids
5	14.42	$[M+H]^+$	$C_{24}H_{39}NO_9$	485.26	Mesaconine	486.2683; 468.2594; 454.2427; 436.2318; 404.2058	Aconite	Alkaloids
6	14.98	$[M+H]^+$	$C_{22}H_{35}NO_4$	377.26	Carmichaeline	378.2650; 360.2541; 342.2438; 328.2284	Aconite	Alkaloids
7	15.21	$[M+H]^+$	$C_{23}H_{37}NO_5$	407.27	Talatizidine	408.2768; 390.2647; 358.2387	Aconite	Alkaloids
8	15.64	$[M+H]^+$	$C_{22}H_{31}NO_3$	357.23	Songorine	358.2395; 340.2283	Aconite	Alkaloids
9	15.75	$[M-H]^-$	$C_{11}H_{12}O_6$	240.06	Eucomic acid	239.0590; 179.0369; 149.0621; 133.0670; 107.0508	Aconite	Phenolic acids
10	15.89	$[M+H]^+$	$C_{25}H_{41}NO_9$	499.28	Aconine	500.2893; 482.2765; 468.2612; 450.2526; 418.2290	Aconite	Alkaloids
11	17.16	$[M+H]^+$	$C_{24}H_{39}NO_7$	453.27	Fuziline	454.2823; 436.2720; 404.2447	Aconite	Alkaloids
12	17.99	$[M+H]^+$	$C_{24}H_{39}NO_6$	437.28	Neoline	438.2876; 420.2775; 388.2486; 154.1229	Aconite	Alkaloids
13	19.86	$[M+H]^+$	$C_{24}H_{39}NO_5$	421.28	Talatisamine	422.2917; 390.2639; 358.2378	Aconite	Alkaloids
14	22.63	$[M+FA-H]^-$	$C_{42}H_{74}O_{15}$	818.50	(3β,6β,12β)-3,12,25-Trihydroxydammarane-6,20-diylbis[β-D-glucopyranoside]	863.4933; 817.4885; 655.4389; 493.3861; 179.0550	Ginseng	Triterpene saponins
15	23.59	$[M+H]^+$	$C_{31}H_{43}NO_{11}$	605.28	14-Benzoyl-10-hydroxymesaconine	606.2953; 588.2840; 574.2667; 556.2552; 524.2336	Aconite	Alkaloids
16	26.22	$[M+H]^+$	$C_{32}H_{45}NO_{11}$	619.31	14-Benzoyl-10-hydroxyaconine	620.3112; 602.2981; 588.2822; 570.2726; 538.2437	Aconite	Alkaloids
17	28.42	$[M+H]^+$	$C_{31}H_{43}NO_{10}$	589.29	Benzoylmesaconine	590.2986; 558.2716; 540.2589; 508.2348	Aconite	Alkaloids
18	31.32	$[M+H]^+$	$C_{32}H_{45}NO_{10}$	603.30	Benzoylaconine	604.3156; 586.3002; 572.2890; 554.2755; 540.2642	Aconite	Alkaloids
19	33.15	$[M+H]^+$	$C_{31}H_{43}NO_9$	573.29	Benzoylhypaconine	574.3052; 542.2782; 510.2519	Aconite	Alkaloids
20	33.56	$[M+FA-H]^-$	$C_{42}H_{72}O_{14}$	800.49	Ginsenoside Rg1	845.4898; 799.4834; 637.4312; 619.4217; 475.3793	Ginseng	Triterpene saponins
21	33.75	$[M+FA-H]^-$	$C_{48}H_{82}O_{18}$	946.55	Ginsenoside Re	991.5563; 945.5425; 799.4886; 637.4337	Ginseng	Triterpene saponins
22	34.91	$[M+H]^+$	$C_{32}H_{45}NO_9$	587.31	14-Benzoyldeoxyaconine	588.3237; 556.2935; 524.2674	Aconite	Alkaloids
23	37.46	$[M+FA-H]^-$	$C_{42}H_{72}O_{14}$	800.49	Ginsenoside Rf	845.4950; 799.4862; 637.4370; 475.3815	Ginseng	Triterpene saponins

24	38.05	$[M+H]^+$	$C_{32}H_{45}NO_8$	571.31	14-O-Anisoylneo-line	572.3264; 540.2989	Aconite	Alkaloids
25	38.19	$[M+H]^+$	$C_{33}H_{45}NO_{10}$	615.30	Hypaconitine	616.3156; 556.2935; 524.2655; 338.1758	Aconite	Alkaloids
26	38.33	$[M+FA-H]^-$	$C_{41}H_{70}O_{13}$	770.48	Ginsenoside F5	815.4807; 769.4687; 637.4266; 475.3766	Ginseng	Triterpene saponins
27	39.50	$[M+FA-H]^-$	$C_{36}H_{62}O_9$	638.44	Ginsenoside F1	683.4373; 637.4330; 475.3807; 161.0463	Ginseng	Triterpene saponins
28	39.86	$[M+FA-H]^-$	$C_{42}H_{72}O_{13}$	784.50	Ginsenoside Rg2	829.4940; 783.4884; 637.4328; 475.3781	Ginseng	Triterpene saponins
29	40.30	$[M+FA-H]^-$	$C_{36}H_{62}O_9$	638.44	Ginsenoside Rh1	683.4352; 637.4302; 475.3766; 161.0456	Ginseng	Triterpene saponins
30	41.13	$[M+FA-H]^-$	$C_{54}H_{92}O_{23}$	1108.60	Ginsenoside Rb1	1153.6062; 1107.5923; 945.5460	Ginseng	Triterpene saponins
31	41.75	$[M-H]^-$	$C_{48}H_{76}O_{19}$	956.50	Ginsenoside Ro	955.4838; 793.4350; 731.4326	Ginseng	Triterpene saponins
32	41.93	$[M+FA-H]^-$	$C_{53}H_{90}O_{22}$	1078.59	Ginsenoside Rc	1123.5860; 1077.5750; 945.5409	Ginseng	Triterpene saponins
33	42.70	$[M+FA-H]^-$	$C_{53}H_{90}O_{22}$	1078.59	Ginsenoside Rb2	1123.5956; 1077.5739; 945.5345	Ginseng	Triterpene saponins
34	42.93	$[M+FA-H]^-$	$C_{53}H_{90}O_{22}$	1078.59	Ginsenoside Rb3	1123.5911; 1077.5801; 945.5391	Ginseng	Triterpene saponins
35	43.56	$[M+FA-H]^-$	$C_{56}H_{94}O_{24}$	1150.61	Quinquenoside R1	1195.6098; 1149.5988; 1107.5815; 1089.5709	Ginseng	Triterpene saponins
36	43.84	$[M-H]^-$	$C_{42}H_{66}O_{14}$	794.45	Chikusetsusapo-nin-Iva	793.4388; 631.3884; 569.3900	Ginseng	Triterpene saponins
37	44.06	$[M+FA-H]^-$	$C_{48}H_{82}O_{18}$	946.55	Ginsenoside Rd	991.5449; 945.5377; 783.4874; 621.4346	Ginseng	Triterpene saponins
38	46.63	$[M+FA-H]^-$	$C_{42}H_{70}O_{12}$	766.49	Ginsenoside Rg6	811.4873; 765.4789; 619.4242; 161.0464	Ginseng	Triterpene saponins
39	47.09	$[M+FA-H]^-$	$C_{42}H_{70}O_{12}$	766.49	Ginsenoside F4	811.4873; 765.4809; 619.4261; 161.0472	Ginseng	Triterpene saponins
40	47.24	$[M+FA-H]^-$	$C_{36}H_{60}O_8$	620.43	Ginsenoside Rk3	665.4270; 619.4225; 161.0466	Ginseng	Triterpene saponins
41	47.84	$[M+FA-H]^-$	$C_{36}H_{60}O_8$	620.43	Ginsenoside Rh4	665.4265; 619.4208; 161.0459	Ginseng	Triterpene saponins
42	48.64	$[M-H]^-$	$C_{42}H_{66}O_{14}$	794.45	Zingibroside R1	793.4356; 731.4315; 613.3759; 569.3806; 523.3807; 455.3534	Ginseng	Triterpene saponins
43	49.94	$[M+FA-H]^-$	$C_{42}H_{72}O_{13}$	784.50	20(S)-Ginseno-side Rg3	829.4921; 783.4871; 621.4381; 459.3845	Ginseng	Triterpene saponins
44	50.24	$[M+FA-H]^-$	$C_{42}H_{72}O_{13}$	784.50	20(R)-Ginseno-side Rg3	829.5001; 783.4895; 621.4385; 459.3850	Ginseng	Triterpene saponins
45	51.56	$[M+FA-H]^-$	$C_{44}H_{74}O_{14}$	826.51	Ginsenoside Rs3	871.5068; 825.4986; 783.4886; 765.4782; 621.4372; 459.3838	Ginseng	Triterpene saponins
46	51.76	$[M+FA-H]^-$	$C_{44}H_{74}O_{14}$	826.51	20(R)-Ginseno-side Rs3	871.5074; 825.4975; 783.4864; 765.4770; 621.4337; 459.3823	Ginseng	Triterpene saponins
47	52.24	$[M+FA-H]^-$	$C_{42}H_{70}O_{12}$	766.49	Ginsenoside Rg5	811.4858; 765.4793; 603.4297; 161.0465	Ginseng	Triterpene saponins
48	52.41	$[M+FA-H]^-$	$C_{42}H_{70}O_{12}$	766.49	Ginsenoside Rk1	811.4775; 765.4728; 603.4248; 161.0456	Ginseng	Triterpene saponins
49	53.39	$[M+FA-H]^-$	$C_{44}H_{72}O_{13}$	808.50	Ginsenoside Rs5	853.4999; 807.4924; 765.4841; 747.4739; 603.4314	Ginseng	Triterpene saponins
50	53.58	$[M+FA-H]^-$	$C_{44}H_{72}O_{13}$	808.50	Ginsenoside Rs4	853.4991; 807.4920; 765.4761; 747.4672; 603.4249	Ginseng	Triterpene saponins

Note: SFD - Shenfu Decoction; MS/MS - Tandem Mass Spectrometry.
